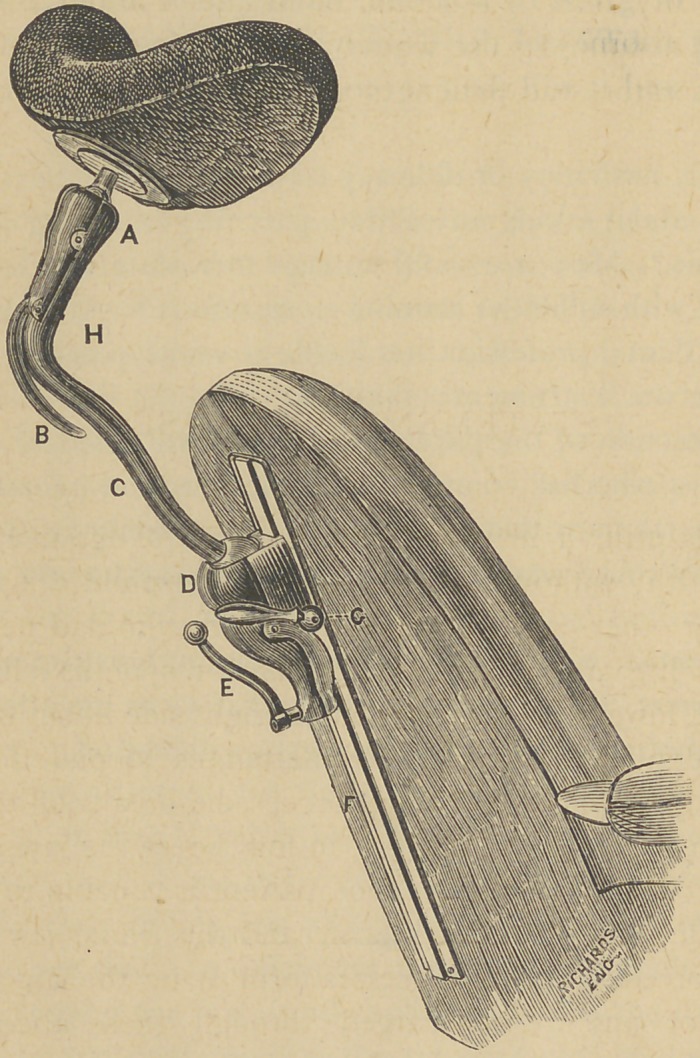# A New Head Rest

**Published:** 1877-05

**Authors:** 


### A NEW HEAD REST.

The accompanying illustration presents a view of a head rest invented and constructed by Dr. T. D. Thompson, of Providence, Rhode Island.

It is so made that it can be easily attached to almost any dental chair, indeed it can be attached to anything where four or six screws can be introduced. It possesses all the movements of the best head rests that has ever been made. It has an upward and downward movement of twenty inches, fixed at any point by the arm G.

The other movements are effected by means of two ball and socket joints; the one at A controlled by the lever B, admits of all the movements to the head piece that could in any case be desired to have. It is released and fastened instantly by means of the lever B.

At D, is another ball and socket joint that admits of movement of the head pad through a circle of twenty inches radius. This joint is fixed and released by the arm E, this by the application of very little power fixes the joint D, immovably.

This rest attached to any ordinary cheap chair, gives it almost the value of those far more expensive. We have never seen anything comparable to this rest, as an appliance adapted to any chair. It should be in the market and attainable at any depot. Its use would afford great comfort to many a weary operator, and patient too.

## Figures and Tables

**Figure f1:**